# Comprehensive Analysis of the Prognostic Values of the TRIM Family in Hepatocellular Carcinoma

**DOI:** 10.3389/fonc.2021.767644

**Published:** 2021-12-23

**Authors:** Weiyu Dai, Jing Wang, Zhi Wang, Yizhi Xiao, Jiaying Li, Linjie Hong, Miaomiao Pei, Jieming Zhang, Ping Yang, Xiaosheng Wu, Weimei Tang, Xiaoling Jiang, Ping Jiang, Li Xiang, Aimin Li, Jianjiao Lin, Side Liu, Jide Wang

**Affiliations:** ^1^ Guangdong Provincial Key Laboratory of Gastroenterology, Department of Gastroenterology, Nanfang Hospital, Southern Medical University, Guangzhou, China; ^2^ Department of Pathology, School of Basic Medical Sciences, Southern Medical University, Guangzhou, China; ^3^ Department of Gastroenterology, Longgang District People’s Hospital, Shenzhen, China

**Keywords:** hepatocellular carcinoma, TRIM family, signature, prognosis, LASSO

## Abstract

**Background:**

Accumulating studies have demonstrated the abnormal expressions and prognostic values of certain members of the tripartite motif (TRIM) family in diverse cancers. However, comprehensive prognostic values of the TRIM family in hepatocellular carcinoma (HCC) are yet to be clearly defined.

**Methods:**

The prognostic values of the TRIM family were evaluated by survival analysis and univariate Cox regression analysis based on gene expression data and clinical data of HCC from The Cancer Genome Atlas (TCGA) and Gene Expression Omnibus (GEO) databases. The expression profiles, protein–protein interaction among the TRIM family, prediction of transcription factors (TFs) or miRNAs, genetic alterations, correlations with the hallmarks of cancer and immune infiltrates, and pathway enrichment analysis were explored by multiple public databases. Further, a TRIM family gene-based signature for predicting overall survival (OS) in HCC was built by using the least absolute shrinkage and selection operator (LASSO) regression. TCGA–Liver Hepatocellular Carcinoma (LIHC) cohort was used as the training set, and GSE76427 was used for external validation. Time-dependent receiver operating characteristic (ROC) and survival analysis were used to estimate the signature. Finally, a nomogram combining the TRIM family risk score and clinical parameters was established.

**Results:**

High expressions of TRIM family members including TRIM3, TRIM5, MID1, TRIM21, TRIM27, TRIM32, TRIM44, TRIM47, and TRIM72 were significantly associated with HCC patients’ poor OS. A novel TRIM family gene-based signature (including TRIM5, MID1, TRIM21, TRIM32, TRIM44, and TRIM47) was built for OS prediction in HCC. ROC curves suggested the signature’s good performance in OS prediction. HCC patients in the high-risk group had poorer OS than the low-risk patients based on the signature. A nomogram integrating the TRIM family risk score, age, and TNM stage was established. The ROC curves suggested that the signature presented better discrimination than the similar model without the TRIM family risk score.

**Conclusion:**

Our study identified the potential application values of the TRIM family for outcome prediction in HCC.

## Introduction

Liver cancer is ranked as the sixth most common malignancy and is the fourth leading cause of cancer-related deaths with an estimated 841,000 new cases and 782,000 deaths occurring worldwide in 2018 (1). Hepatocellular carcinoma (HCC), the major histological type of liver cancer, is related to well-known risk factors, including hepatitis virus (B and C) infections, aflatoxin B1 exposure, alcohol abuse, and smoking, etc (2). Regretfully, less than 40% of HCC patients are eligible for surgery because of advanced stages of HCC at the time of diagnosis (3). Moreover, though the selected HCC patients have received surgical resection, their prognoses remain unsatisfactory due to the high incidence of recurrence (4, 5). Predicting the prognosis accurately may be of use for choosing effective personalized treatment, hence prolonging the survival time for patients with HCC. Therefore, identification of novel biomarkers for outcome prediction is of great importance for HCC.

The tripartite motif (TRIM) family proteins, also named RBBC proteins, are defined by the presence of the TRIM composed of a RING domain, one or two B-box motifs, and a coiled-coil region (6, 7). Most of them process E3 ubiquitin ligase activities because of a RING domain at the N-terminus (8). Accumulating studies have suggested that the TRIM family proteins play important roles in cell proliferation (9, 10), migration and invasion (9–11), metabolism (12), autophagy, immunity, and carcinogenesis (13), etc. A study in prostate cancer showed that collaborating with the AR signaling, TRIM24 could act as a transcriptional activator of genes involved in the progression of the cell cycle (14). It was indicated that AMP-activated protein kinase (AMPK) could be suppressed through its ubiquitination and degradation by the MAGE-A3/6-TRIM28 ubiquitin ligase complex (15). TRIM29 could serve as a scaffold protein for assembling DNA repair proteins into chromatin for efficient DDR activation (16). TRIM32 might target GLUT1 and HKII to mediate the metabolism of glycolysis in gastric cancer cells (12). What is more, the abnormal expressions of many TRIM family proteins have been reported to be a prognostic factor in cancer (17–21). In HCC, Liu et al. found that TRIM25 could directly target Keap1 and promote its ubiquitination and degradation, activating Nrf2 and bolstering antioxidant defense and cell survival (22). A study reported that by directly binding with SNAIL and inducing its K-48 linked poly-ubiquitous degradation, TRIM50 could reverse SNAIL-mediated epithelial-to-mesenchymal transition (EMT) (23) in HCC. It has been demonstrated that the TIF1-related subfamily of TRIM proteins could interact to create regulatory complexes and mediate HCC in mice (24). These studies suggested the important roles of TRIM family proteins in HCC progression. However, the potential application values of the TRIM family for outcome prediction in HCC remain poorly investigated.

In this study, investigations of survival analysis, expression profiles, correlation analysis between the TRIM family and hallmarks of cancer, etc., were performed based on the gene expression and clinical data of HCC from The Cancer Genome Atlas (TCGA) and Gene Expression Omnibus (GEO). Then, a risk score model was constructed by using the least absolute shrinkage and selection operator (LASSO) regression with moderate accuracy. Finally, to improve the predictive value of the TRIM family for HCC patients, a nomogram was built by integrating clinical parameters and the risk score.

## Materials and Methods

### Data Acquisition

The RNA-Seq and clinical information of TCGA–Liver Hepatocellular Carcinoma (LIHC) cohort, which included 50 normal control samples and 374 HCC samples, were downloaded from the Genomic Data Commons Data Portal (GDC; https://portal.gdc.cancer.gov/). The workflow type of transcriptome profiling of TCGA-LIHC was “HTSeq-FPKM”, and the format of the clinical data was “bcr xml”. The gene expression and clinical data of GSE76427 were obtained from GEO (https://www.ncbi.nlm.nih.gov/geo/) for external validation, including 52 normal liver tissue samples and 115 HCC samples (25). GSE76427 was performed on the GPL10558 platform (Illumina HumanHT-12 V4.0 expression beadchip). The gene list of the TRIM family was derived from HUGO Gene Nomenclature Committee (HGNC) database (https://www.genenames.org/). All the data were downloaded in April 2020.

### Univariate Cox Regression Analysis and Survival Analysis

Univariate Cox regression was applied to investigate the prognostic value of reported HCC prognostic biomarkers and the TRIM family members in HCC by using the R package “survival” (26, 27). The Kaplan–Meier curve with a two-sided log-rank test was analyzed to evaluate whether the TRIM family members or the signature was associated with the outcome of patients with HCC. X-tile was used to investigate the optimal cutoff value for survival analysis (28).

### Analysis of the Expression of the TRIM Family

The expression profiles of the TRIM family were presented by using the R package “pheatmap” (29). The expressions of the TRIM family members between tumor and normal samples from TCGA and GEO cohorts were shown by boxplots. The correlations among the TRIM family or the correlations between the TRIM family and several molecular markers relevant to the hallmarks of cancer were plotted with the R package “corrplot” (30), and they were analyzed by Spearman’s test.

### Cell Culture

Human HCC cell lines HepG2, Huh7, Hep3B, and SK-Hep-1 were purchased from Procell (Wuhan, China). MHCC97-H was acquired from GuangZhou Jennio Biotech Co., Ltd. (Guangzhou, China). A human normal hepatocyte line MIHA was purchased from Shanghai Honsun Biological Technology Co., Ltd. (Shanghai, China). Human HCC cell lines HepG2, Huh7, MHCC97H, Hep3B, and SK-Hep-1 and human normal hepatocyte cell line MIHA were cultured using Dulbecco’s modified Eagle’s medium (DMEM; Gibco, Carlsbad, CA, USA) supplemented with 10% fetal bovine serum (FBS; Gibco, Carlsbad, CA, USA) at 37°C in 5% CO_2_.

### Patients and Tissue Samples

The liver tissue samples including 38 HCC samples and paired adjacent normal tissue samples were obtained from HCC patients receiving surgical resection in Nanfang Hospital, Southern Medical University (Guangzhou, China). None of the patients underwent chemotherapy, radiotherapy, or immunotherapy before surgery. The experiments conducted in this study were approved by the Medical Ethics Committee of Nanfang Hospital, Southern Medical University.

### RNA Isolation and Quantitative Real-Time PCR

Total RNAs from cultured cells, HCC samples, and adjacent normal liver tissue samples were extracted using TRIzol reagent (Invitrogen, Carlsbad, CA, USA). To detect the mRNA expression, a qRT-PCR assay was carried out using the PrimeScript RT Reagent Kit (Takara Bio, Inc., Shiga, Japan) and SYBR Premix Ex Taq (Takara Bio, Inc., Shiga, Japan) according to the manufacturer’s instructions. GAPDH was chosen as the endogenous control. The final data were calculated using the 2^−ΔΔCt^ method. The primer sequences are listed in **Supplementary Table 1**.

### Public Database Mining

Expressions of the TRIM family members in both HCC and normal liver tissue at the protein level were retrieved from the Human Protein Atlas (https://www.proteinatlas.org/) (31, 32). The co-expressed genes of the TRIM family members with Spearman’s correlation ≥0.3 or <−0.3 were retrieved from the cBioportal database (33), and they were annotated by the Kyoto Encyclopedia of Genes and Genomes (KEGG) pathway. The KEGG (34) enrichment analysis was conducted with the package “clusterProfiler” (35) and visualized with package “enrichplot” (36) in R. The enrichment results with *p*-value <0.05 and q-value <0.05 were considered statistically significant. The cBioPortal database was used to explore the genetic alterations of the TRIM family members. Protein–protein interaction of the TRIM family members was analyzed by STRING database (minimum required interaction score = 0.150, https://string-db.org/) (37). NetworkAnalyst (https://www.networkanalyst.ca/) (38) was applied to predict the potential transcription factors (TFs) and miRNAs of the TRIM family members, and the results were presented by using Cytoscape 3.7.0 (39). The potential TFs were retrieved from the ENCODE database (40) *via* the NetworkAnalyst platform, while the predictions of miRNAs were based on the TarBase database (41). The correlations between TRIM family and immune infiltrates including B cells, CD8+ T cells, CD4+ T cells, macrophages, neutrophils, and dendritic cells were analyzed *via* the TIMER (Tumor IMmune Estimation Resource, https://cistrome.shinyapps.io/timer/) platform (42), shown by the purity-corrected partial Spearman method.

### Construction of the TRIM Family Gene-Based Signature

The LASSO regression was applied to construct the prognostic with the R package “glmnet” (43) based on lambda.min. The optimal tuning parameter (lambda) was determined through tenfold cross-validations. To calculate the risk score, the expression of each gene in the signature was multiplied by its regression coefficient, and then these values were summed. Survival analysis was applied to assess the predictive value of the signature. Time-dependent receiver operating characteristic (ROC) curve was performed to calculate the area under the curve (AUC) for 1-, 3-, or 5-year overall survival (OS) and to evaluate the discrimination of the signature by using the R package “survivalROC” (44). Univariate and multivariate Cox regression analyses were performed by using the R package “survival” to investigate whether the risk score was an independent OS predictor for patients with HCC.

### Correlation Between the Signature and Clinical Factors

To confirm the predictive value of the signature, patients were stratified into different subgroups according to age, gender, TNM stage, T stage, N stage, M stage, or BCLC stage; and survival analysis was performed in each subgroup. Boxplots were applied to present the risk score in different subgroups. The chi-square test or Fisher’s exact test was used to analyze the relationship between the risk score levels and clinical parameters.

### Gene Set Enrichment Analysis

Differences of KEGG pathways between high- and low-risk groups stratified based on the signature were analyzed by using GESA (v4.0.3, https://www.gsea-msigdb.org/gsea/index.jsp) (45). Enrichment results with a *p*-value <0.05 and a false discovery rate (FDR) <0.25 after performing 1,000 permutations were considered statistically significant. c2.cp.kegg.v7.0.symbols.gmt was selected as the reference set for calculating Enrichment Score (ES). The results were plotted with R package “ggplot2” (46).

### Establishment of the Nomogram

Combining the risk score and some clinical factors, a nomogram was built by using the R package “rms” (47). C-index, time-dependent ROC curve, and calibration curve were applied to evaluate the discrimination and calibration of the nomogram. The calibration curve was performed to observe whether the predicted survival outcome was close to the actual outcome by a bootstrap method with 1,000 resamples.

### Statistical Analysis

All statistical analyses were conducted with R software version 3.6.0 (The R Foundation for Statistical Computing, Vienna, Austria), IBM SPSS Statistics 23 (SPSS, Inc., Chicago, IL, USA), and GraphPad Prism software version 7.0 (GraphPad Software Inc. USA). The statistical significance of boxplots was analyzed by the Mann–Whitney U test. The differences in group comparisons from qRT-PCR results were assessed using a two-tailed Student’s t-test or one-way ANOVA. The qRT-PCR results were shown as mean ± SD. A *p*-value <0.05 was considered statistically significant.

## Results

### The Prognostic Values and Expression Profiles of the TRIM Family

**Figure 1:**
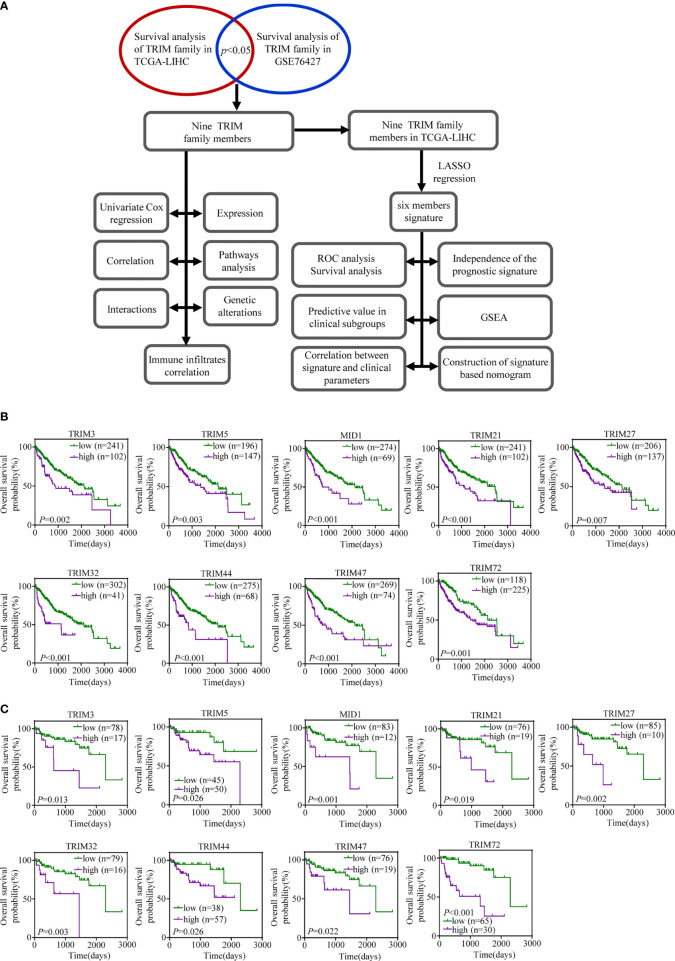
The prognostic values of the nine TRIM family members in HCC. **(A)** The workflow of our analysis steps. **(B, C)** Kaplan–Meier estimates of OS based on the nine TRIM family members in TCGA-LIHC **(B)** and GSE76427 **(C)**. TRIM, tripartite motif; HCC, hepatocellular carcinoma; OS, overall survival; TCGA-LIHC, The Cancer Genome Atlas Liver Hepatocellular Carcinoma.

**Figure 2:**
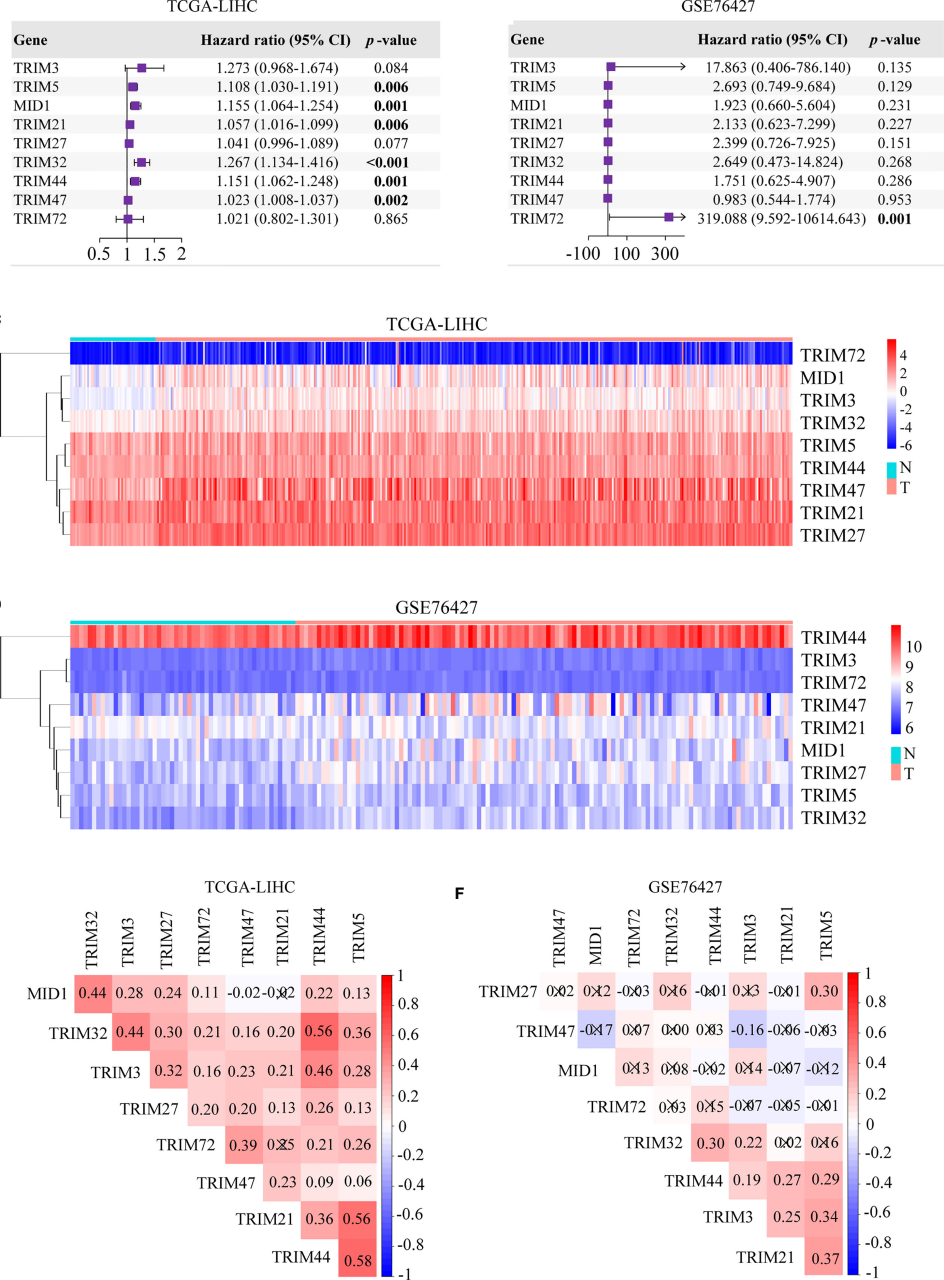
Univariate Cox regression analyses and the heatmaps of nine TRIM family members in HCC. **(A, B)** Univariate Cox regression analyses of nine TRIM family members in TCGA-LIHC **(A)** and GSE76427 **(B)**. **(C, D)** The heatmaps depict the expression profiles of nine TRIM family members in TCGA-LIHC **(C)** and GSE76427 **(D)**. **(E, F)** Correlation plots of nine TRIM family members in TCGA-LIHC **(E)** and GSE76427 **(F)**. TRIM, tripartite motif; HCC, hepatocellular carcinoma; TCGA-LIHC, The Cancer Genome Atlas Liver Hepatocellular Carcinoma.

Our study was conducted as illustrated in **Figure 1A**. A total of 343 patients in TCGA-LIHC and 95 patients in GSE76427 with survival time of no less than 30 days were included for survival analysis. All genes in the TRIM family were subjected to the analysis, and then nine genes including TRIM3, TRIM5, MID1, TRIM21, TRIM27, TRIM32, TRIM44, TRIM47, and TRIM72 were identified as genes that might be associated with HCC patients’ OS in both TCGA-LIHC and GSE76427 (*p* < 0.05, **Figures 1B, C**). Moreover, we applied univariate Cox regression analyses to explore prognostic values of five reported HCC prognostic biomarkers and nine TRIM family members in HCC (**Supplementary Figures 1A, B** and **Figures 2A, B**) (48–52). The results showed that five recognized prognostic biomarkers (PDCD10, TFAP4, LYRM4, VPS35, and PPM1D) were prognosis-associated in TCGA-LIHC, and VPS35 was related to prognosis in both TCGA-LIHC and GSE76427 with an HR > 1 (*p* < 0.05). Six genes (TRIM5, MID1, TRIM21, TRIM32, TRIM44, and TRIM47) were prognosis-associated in TCGA-LIHC, while only one gene (TRIM72) was relevant to prognosis in GSE76427 with an HR > 1 among the nine TRIM family members (*p* < 0.05), which was similar to results of the five reported HCC prognostic biomarkers. The expression profiles of the nine TRIM family members in TCGA-LIHC and GSE76427 are shown in **Figures 2C, D**. The correlations among the nine TRIM family members in TCGA-LIHC and GSE76427 are presented in **Figures 2E, F**.

### Expression Patterns, Pathway Analysis, and Genetic Alterations of the TRIM Family

**Figure 3:**
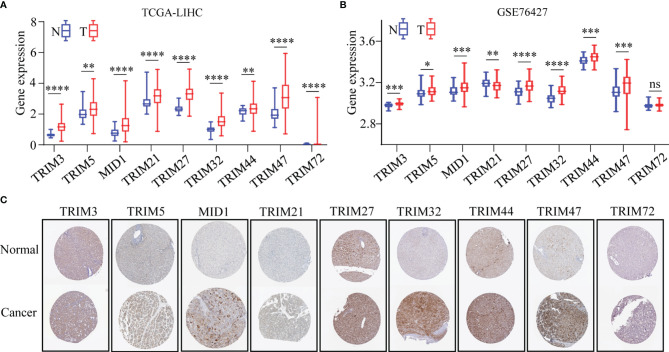
The expression profiles of the nine TRIM family members in HCC. **(A, B)** The expressions of nine TRIM family members in TCGA-LIHC **(A)** and GSE76427 **(B)**. **(C)** Expressions at the protein level of nine TRIM family members *via* the Human Protein Atlas database. ns, no significance; *p < 0.05; **p < 0.01; ***p < 0.001; ****p < 0.0001. TRIM, tripartite motif; HCC, hepatocellular carcinoma; TCGA-LIHC, The Cancer Genome Atlas Liver Hepatocellular Carcinoma.

The differential expressions of the nine genes between normal tissues and HCC tissues were investigated. It was found that eight genes consisting of TRIM3, TRIM5, MID1, TRIM21, TRIM27, TRIM32, TRIM44, and TRIM47 were upregulated in HCC with significant differentiation in TCGA-LIHC and GSE76427 cohorts (*p* < 0.05, **Figures 3A, B**). The expressions at a translational level of the nine genes were shown by the immunohistochemistry (IHC) staining images from The Human Protein Atlas database (**Figure 3C**).

**Figure 4:**
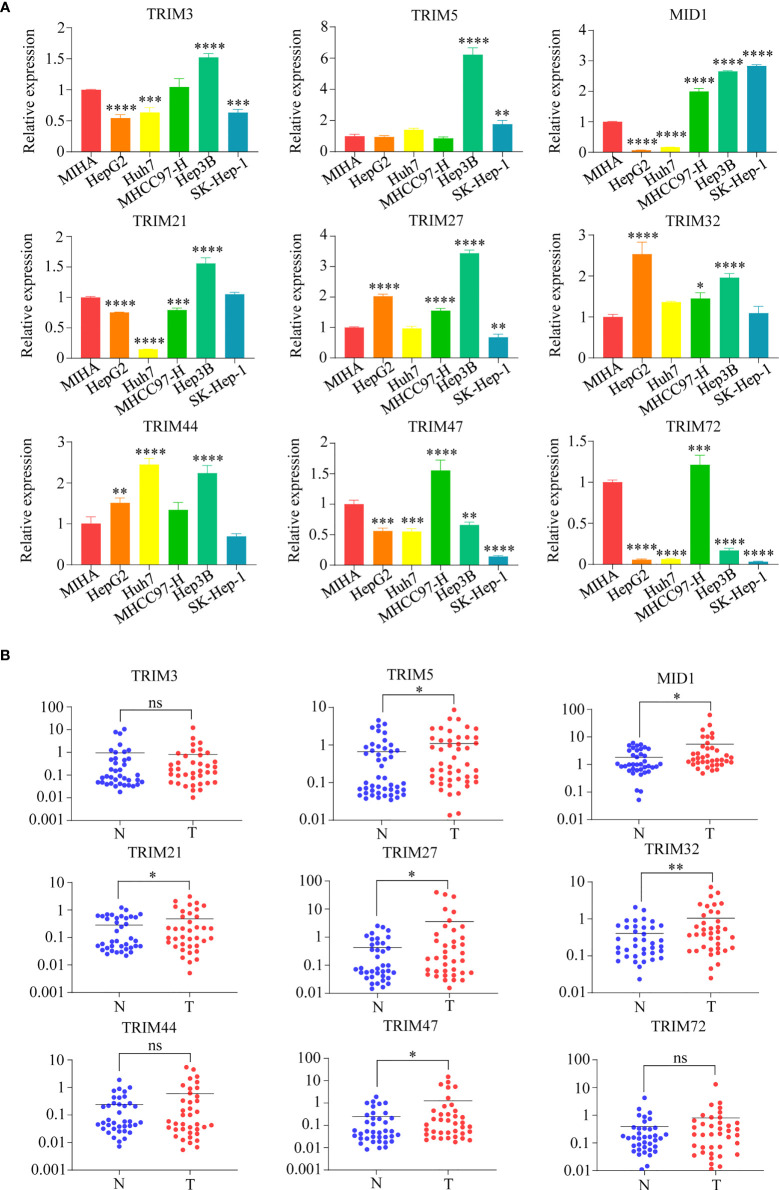
The mRNA expressions of nine TRIM family members (TRIM3, TRIM5, MID1, TRIM21, TRIM27, TRIM32, TRIM44, TRIM47, and TRIM72) in human HCC cell lines and HCC samples. **(A)** The mRNA expressions of nine TRIM family members in human HCC cell lines (HepG2, Huh7, MHCC97H, Hep3B, and SK-Hep-1) and normal human hepatocyte cell line MIHA were measured by qRT-PCR. The normal human liver cell line MIHA was used as a control. **(B)** The mRNA expressions of nine TRIM family members in 38 HCC samples and the corresponding adjacent normal tissue samples. ns, no significance; *p < 0.05; **p < 0.01; ***p < 0.001; ****p < 0.0001. TRIM, tripartite motif; HCC, hepatocellular carcinoma.

Furthermore, the mRNA expression levels of the nine TRIM family members (TRIM3, TRIM5, MID1, TRIM21, TRIM27, TRIM32, TRIM44, TRIM47, and TRIM72) were detected in human HCC cell lines (HepG2, Huh7, MHCC97H, Hep3B, and SK-Hep-1) and normal human hepatocyte cell line MIHA by qRT-PCR assays (**Figure 4A**). The mRNA levels of TRIM5 in Hep3B, MID1 in Hep3B and SK-Hep-1, TRIM27 in HepG2 and Hep3B, TRIM32 in HepG2, and TRIM44 in Huh7 and Hep3B were 2-fold higher than those in MIHA. The mRNA expressions of the nine TRIM family members in 38 HCC samples and corresponding adjacent normal liver tissue samples were investigated by qRT-PCR assays (**Figure 4B**). The results showed that the mRNA levels of TRIM5, MID1, TRIM21, TRIM27, TRIM32, and TRIM47 were significantly upregulated in the HCC tissues.

**Figure 5:**
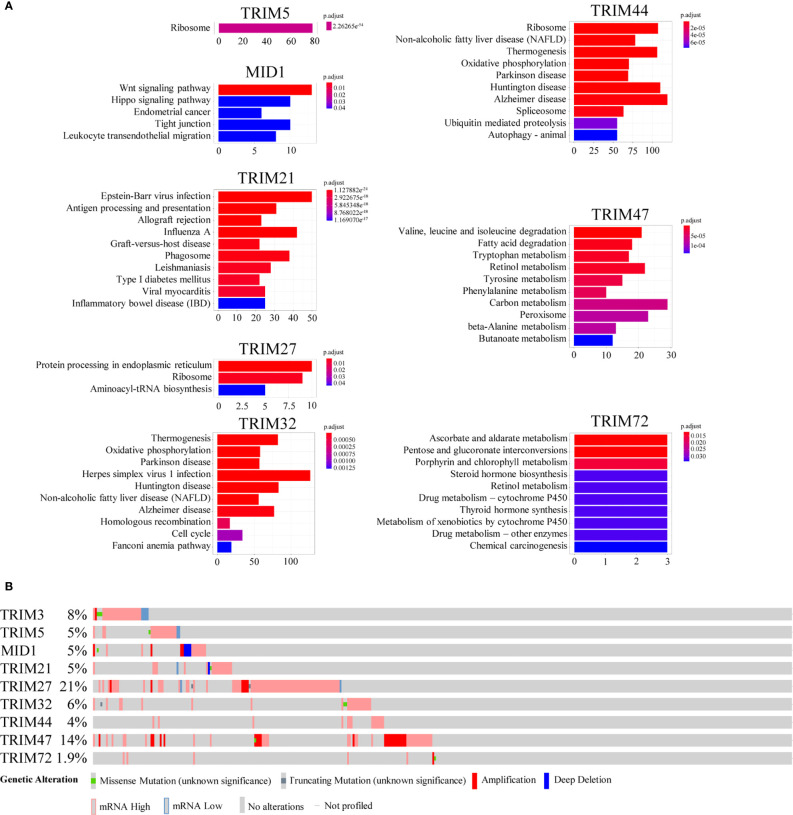
The potential mechanisms of the nine TRIM family members. **(A)** KEGG enrichment analyses for co-expressed genes associated with the nine TRIM family members. **(B)** The genetic alteration profiles of the nine TRIM family members *via* the cBioportal database. TRIM, tripartite motif; KEGG, Kyoto Encyclopedia of Genes and Genomes.

The co-expressed genes of the nine TRIM family members were annotated by the KEGG pathway (**Figure 5A**). Wnt signaling pathway; Epstein–Barr virus infection; protein processing in the endoplasmic reticulum; thermogenesis; valine, leucine, and isoleucine degradation; and ascorbate and aldarate metabolism were the top KEGG pathways of MID1, TRIM21, TRIM27, TRIM32, TRIM47, and TRIM72, respectively. On the other hand, there was no significant term for TRIM3. Ribosome was the top term in KEGG for both TRIM44 and TRIM5. The genetic alterations of the nine TRIM family members explored by using the cBioPortal database ranged from 1.9% to 21% (**Figure 5B**).

### Protein–Protein Interaction, Transcription Factors, and miRNA Predictions of the TRIM Family

**Figure 6:**
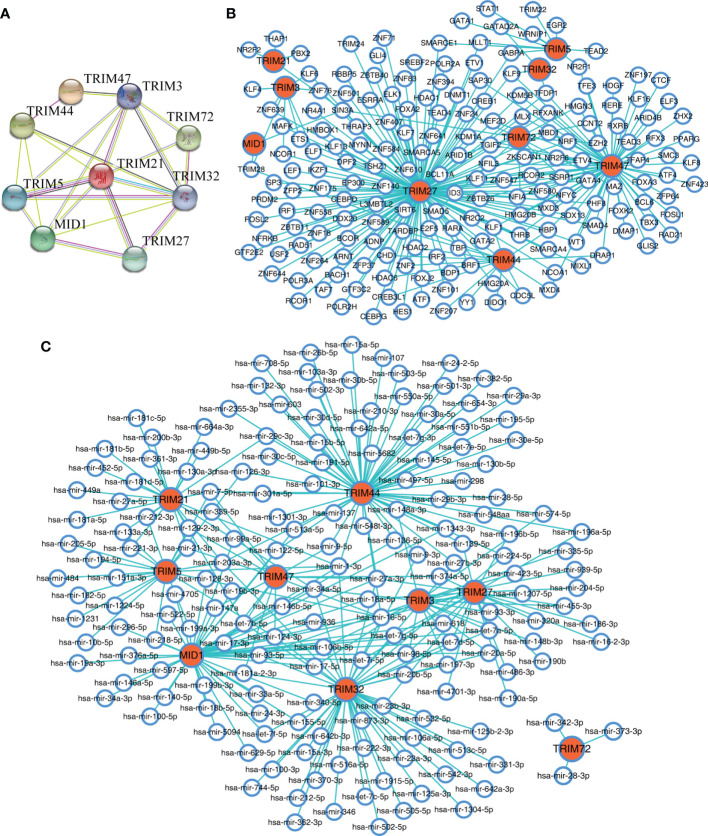
The interaction networks of the nine TRIM family members. **(A)** The protein–protein interaction network of the nine TRIM family members based on the STRING database. **(B, C)** The predicted TFs **(B)** and miRNAs **(C)** connected with the nine TRIM family members in the NetworkAnalyst database. TRIM, tripartite motif; TFs, transcription factors.

The protein–protein interaction network constructed by STRING database suggested that the nine members showed extensive associations with each other at the protein level (**Figure 6A**). With the use of the NetworkAnalyst database, the predicted TFs and miRNAs that connected with the nine members are shown in **Figures 6B, C**.

### Correlations With the Hallmarks of Cancer of the Nine TRIM Family Members

**Figure 7:**
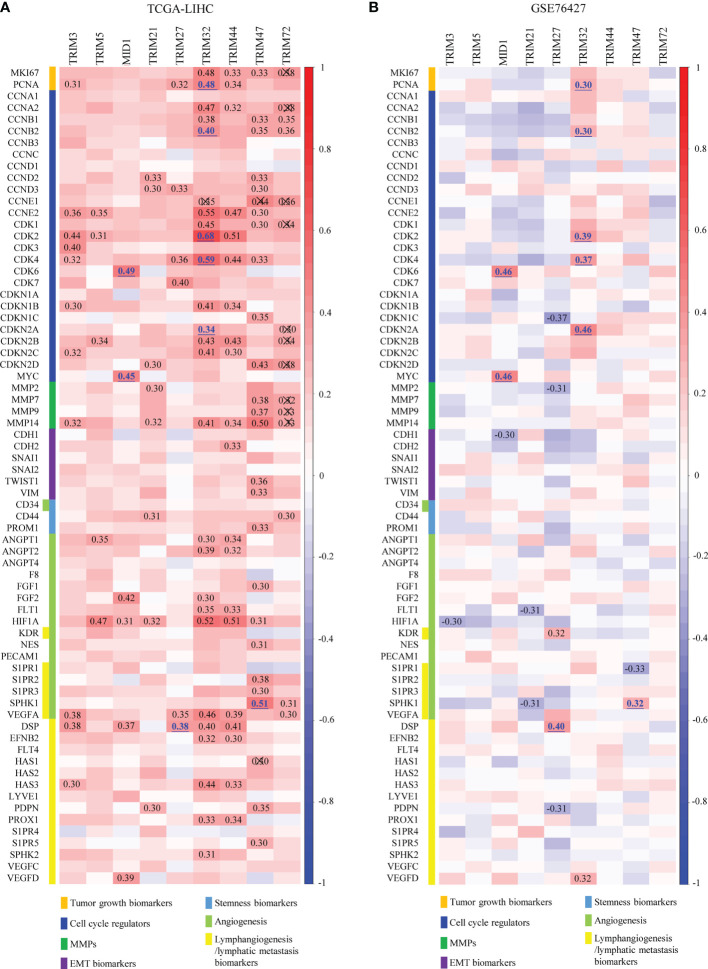
Overall correlations between the expressions of the nine TRIM family members and the hallmarks of cancer. **(A, B)** Correlations between the expressions of the nine TRIM family members and several markers of proliferation, cell cycle, invasion/migration, EMT, stemness, angiogenesis, and lymphangiogenesis in TCGA-LIHC **(A)** and GSE76427 **(B)**. TRIM, tripartite motif; EMT, epithelial-to-mesenchymal transition; TCGA-LIHC, The Cancer Genome Atlas Liver Hepatocellular Carcinoma.

To explore the potential mechanisms of the nine TRIM family members in HCC, the correlations between the nine TRIM family members and several markers of proliferation, cell cycle, invasion/migration, EMT, stemness, angiogenesis, and lymphangiogenesis were analyzed (**Figures 7A, B**). TRIM32 was shown to be positively correlated with the proliferation index proliferating cell nuclear antigen (PCNA) and cell cycle markers (CCNB2, CDK2, CDK4, and CDKN2A) in both cohorts, and MID1 exhibited significant positive correlations with CDK6 and MYC in TCGA-LIHC and GSE76427 cohorts. However, though some correlations between TRIM family and the markers of invasion/migration, EMT, and stemness could be observed in TCGA cohort, we could not obtain similar results in the GSE76427 cohort. As regards angiogenesis and lymphangiogenesis, TRIM47 presented positive correlations with SPHK1 in both cohorts. Besides, positive correlations were shown between TRIM27 and the lymphangiogenesis marker DSP in the two cohorts. Thus, the nine TRIM family members may play important roles in cell proliferation, cell cycle, angiogenesis, and lymphangiogenesis.

### TRIM Family Members Associate With Immune Infiltrates

**Figure 8:**
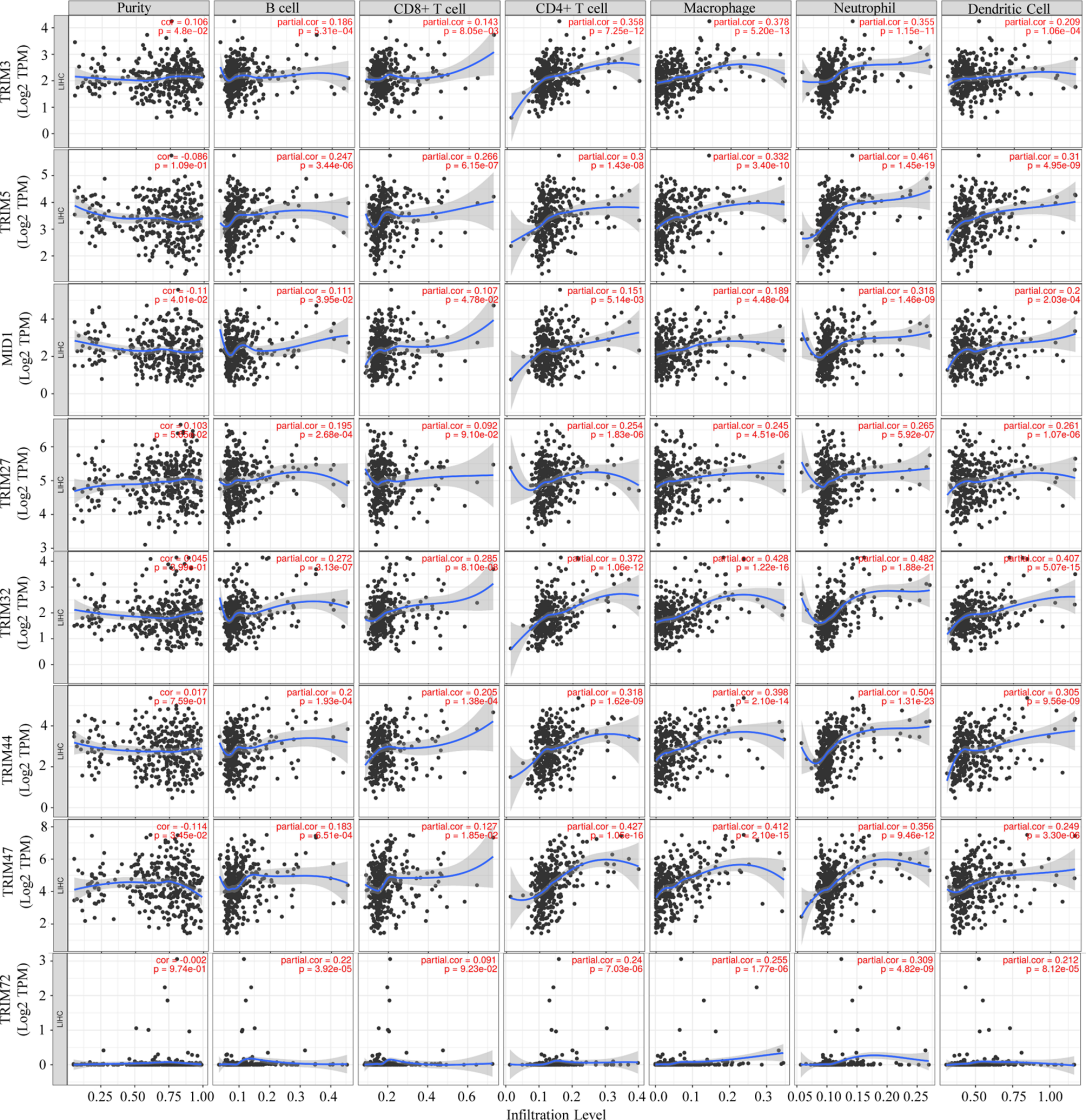
Associations of the nine TRIM family members with tumor immune infiltrating cells (B cells, CD8+ T cells, CD4+ T cells, macrophages, neutrophils, and dendritic cells) *via* the TIMER database. TRIM, tripartite motif; TIMER, Tumor IMmune Estimation Resource.

In order to investigate whether the TRIM family plays potential biological roles in immune infiltrates, the correlations between the nine TRIM family members and the immune infiltration level in LIHC were analyzed based on the TIMER database (**Figure 8**). Intriguingly, positive correlations were identified between TRIM3 and CD4+ T cell (r = 0.358, *p* = 7.25e−12), macrophages (r = 0.378, *p* = 5.20e−13), or neutrophils (r = 0.355, *p* = 1.15e−11). TRIM5 expression exhibited significant associations with CD4+ T cell (r = 0.3, *p* = 1.43e−08), macrophages (r = 0.332, *p* = 3.40e−10), neutrophils (r = 0.461, *p* = 1.45e−19), and dendritic cells (r = 0.31, *p* = 4.95e−09). By using the TIMER database, Qi et al. reported the positive correlations between TRIM21 expression and immune infiltrates, such as B cells, CD4+ T cells, macrophages, neutrophils, and dendritic cells (53). Positive associations were shown between TRIM32 and CD4+ T cell (r = 0.372, *p* = 1.06e−12), macrophages (r = 0.428, *p* = 1.22e−16), neutrophils (r = 0.482, *p* = 1.88e−21), and dendritic cells (r = 0.407, *p* = 5.07e−15). TRIM44 expression was significantly correlated with CD4+ T cell (r = 0.318, *p* = 1.62e−09), macrophages (r = 0.398, *p* = 2.10e−14), neutrophils (r = 0.504, *p* = 1.31e−23), and dendritic cells (r = 0.305, *p* = 9.56e−09). Positive correlations could be found between TRIM47 and CD4+ T cell (r = 0.427, *p* = 1.05e−16), macrophages (r = 0.412, *p* = 2.10e−15), and neutrophils (r = 0.356, *p* = 9.46e−12). Besides, TRIM72 expression showed a significant correlation with neutrophils (r = 0.309, *p* = 4.82e−09).

### Construction and Validation of the TRIM Family Gene-Based Signature

**Figure 9:**
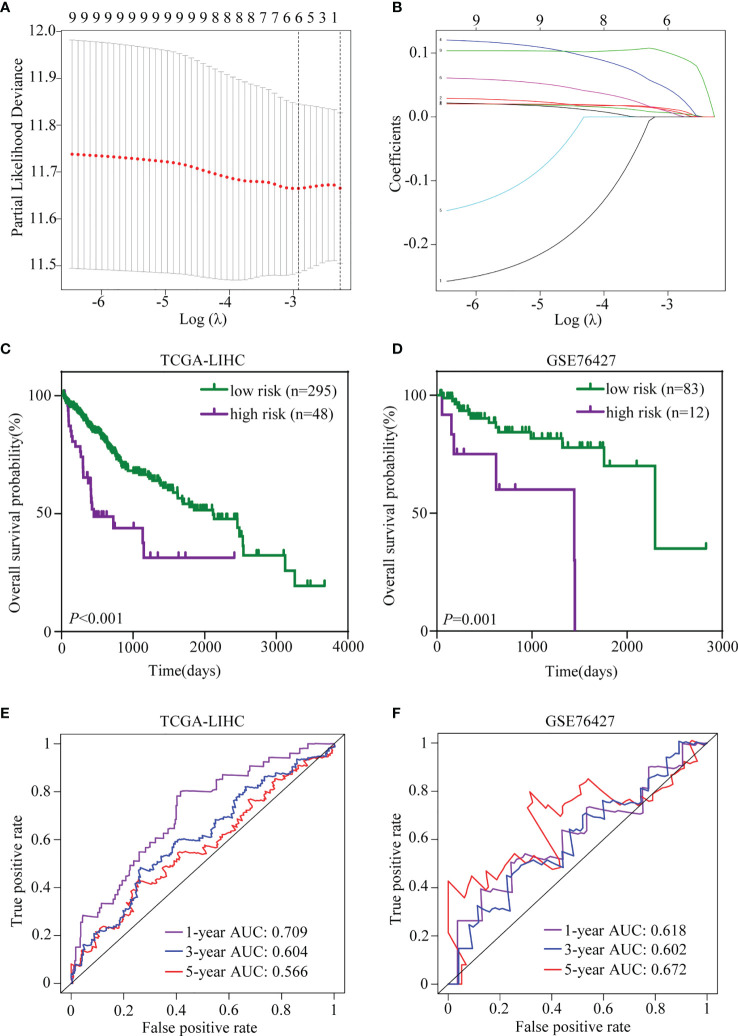
Establishment and validation of the TRIM family gene-based signature. **(A)** Tenfold cross-validation for selecting the optimal tuning parameter (λ) in the LASSO model. **(B)** LASSO coefficient profiles of the nine TRIM family members. **(C, D)** Kaplan–Meier plots of OS for HCC patients based on the TRIM family gene-based signature in TCGA-LIHC **(C)** and GSE76427 **(D)** cohorts. **(E, F)** Time-dependent ROC curves for OS of the TRIM family gene-based signature in TCGA-LIHC **(E)** and GSE76427 **(F)** cohorts. TRIM, tripartite motif; LASSO, least absolute shrinkage and selection operator; OS, overall survival; HCC, hepatocellular carcinoma; TCGA-LIHC, The Cancer Genome Atlas Liver Hepatocellular Carcinoma; ROC, receiver operating characteristic.

Given that more and more attention is being paid to the prognostic value of gene signature, a TRIM family signature was also constructed based on the nine members, which had significant values for OS prediction. TCGA-LIHC dataset (n = 343) was used as the testing set, and GSE76427 (n = 95) was selected for validation. The LASSO regression was applied to reduce multicollinearity among the TRIM family members and select the appropriate members for the signature (**Figures 9A, B**). Therefore, a six-gene-based signature was constructed with their regression coefficients derived from the LASSO regression analysis: risk score = 0.00563719 × Exp_(TRIM5)_ + 0.03899426 × Exp_(MID1)_ + 0.00885929 × Exp_(TRIM21)_ + 0.09782539 × Exp_(TRIM32)_ + 0.01168274 × Exp_(TRIM44)_ + 0.00588728 × Exp_(TRIM47)_. As above, the coefficients of the six members indicated that they were all risk factors for OS, and TRIM32 had the most influence on OS while TRIM5 had the least. The risk score of each patient was calculated based on the formula above. Then, patients were divided into high- and low-risk groups based on the optimal cutoff, which was determined by X-tile software. The profiles of the risk score, survival status, and gene expression levels are shown in **Supplementary Figures 2A, B**. In the training set, the survival analysis manifested that patients in the high-risk group had worse OS than the low-risk patients (**Figure 9C**). Time-dependent ROC analysis showed that the AUCs for 1-, 3-, and 5-year OS were 0.709, 0.604, and 0.566, respectively (**Figure 9E**). Similar results could be obtained in the validation set. The OS of the patients who belonged to the high-risk group was worse than that of the low-risk group (**Figure 9D**). The ROC analysis indicated that the prognostic accuracy of the signature was 0.618 at 1 year, 0.602 at 3 years, and 0.672 at 5 years (**Figure 9F**). Together, the six-gene prognostic signature performed well in OS prediction for HCC patients.

### Univariate and Multivariate Cox Regression Analyses and Subgroup Analyses of the Signature

**Figure 10:**
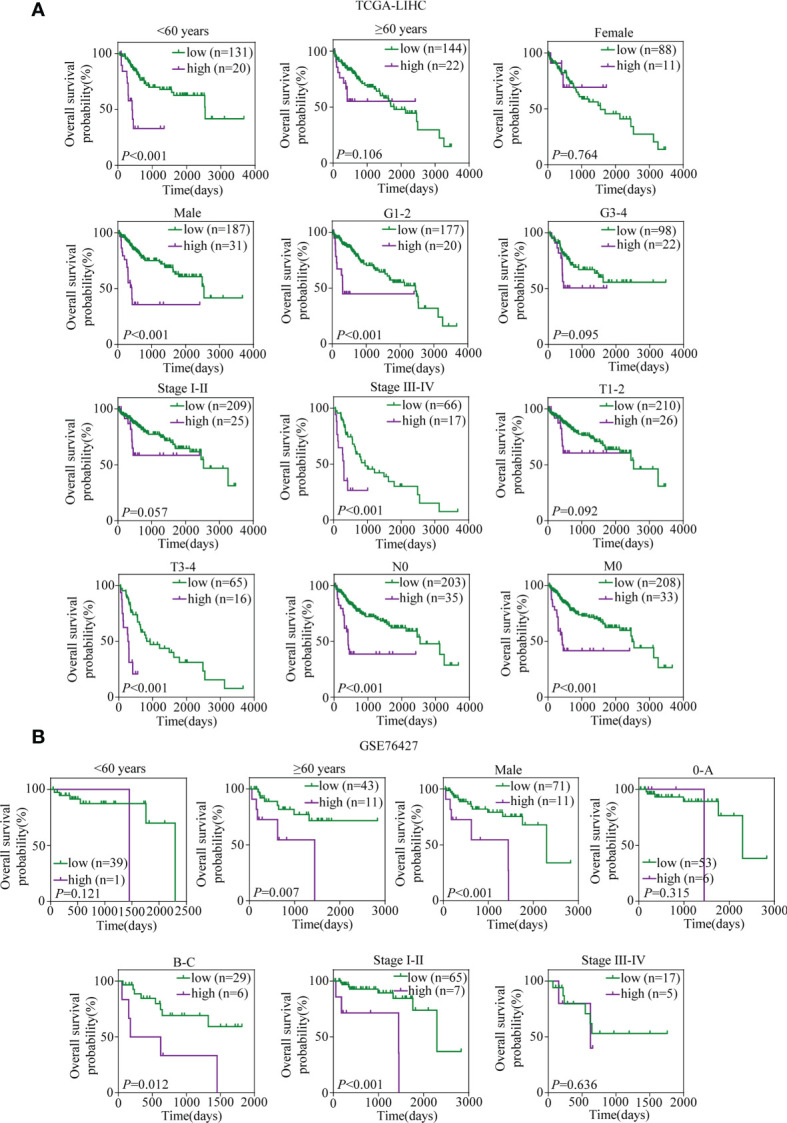
Kaplan–Meier survival analyses for prognostic values of the TRIM family gene-based signature in different subgroups stratified by clinical features. **(A)** Kaplan–Meier curves for OS of the six-TRIM family gene risk score level in subgroups including <60 years, ≥60 years, female, male, G1–2, G3–4, stage I–II, stage III–IV, T1–2, T3–4, N0, and M0 in TCGA-LIHC cohort. **(B)** Kaplan–Meier curves for OS of the six-TRIM family gene risk score level in subgroups including <60 years, ≥60 years, male, 0–A, B–C stage I–II, and stage III–IV in GSE76427. TRIM, tripartite motif; OS, overall survival; TCGA-LIHC, The Cancer Genome Atlas Liver Hepatocellular Carcinoma.

A total of 317 patients in TCGA-LIHC with complete clinical information, including age, gender, grade, TNM stage, T stage, N stage, and M stage, and 94 patients in GSE76427 with sufficient information on age, gender, TNM stage, and BCLC stage were included for subsequent analyses. In TCGA-LIHC cohort, the univariate and multivariate Cox regression analyses suggested that the risk score was an independent predictor for OS (**Table 1**). To investigate whether the prognostic signature was applicable to other clinical parameters, all patients were divided into subgroups according to the clinical parameters. Survival analyses in subgroups indicated that the signature was quite useful in several subgroups such as <60 years (*p* < 0.001), male (*p* < 0.001), G1–2 (*p* < 0.001), TNM stage III–IV (*p* < 0.001), T3–4 (*p* < 0.001), N0 (*p* < 0.001), and M0 (*p* < 0.001) in TCGA-LIHC cohort (**Figure 10A**). Similarly, the signature performed well in subgroups including ≥60 years (*p* = 0.007), male (*p* < 0.001), BCLC stage B–C (*p* = 0.012), and TNM stage I–II (*p* < 0.001) for OS prediction in GSE76427 (**Figure 10B**).

**Table 1:** Univariate and multivariate Cox regression analyses of the TRIM family gene-based signature and clinical parameters in TCGA-LIHC.

Characteristics	Number	Univariate Cox regression		Multivariate Cox regression	
		Hazard ratio (95%CI)	*p*-Value	Hazard ratio (95%CI)	*p*-Value
**Age**	317	1.006 (0.991–1.021)	0.451	1.008 (0.993–1.024)	0.285
**Gender**					
Male/female	218/99	0.796 (0.534–1.189)	0.265		
**Grade**					
G3–4/G1–2	120/197	1.061 (0.711–1.584)	0.771		
**Tumor stage**					
III–IV/II–I	83/234	2.842 (1.925–4.197)	<0.001	2.619 (1.763–3.890)	<0.001
**T**					
T3–4/T1–2	81/236	2.866 (1.939–4.236)	<0.001		
**N**					
N1/N0	3/238	2.186 (0.534–8.950)	0.277		
Nx/N0	76/238	1.204 (0.763–1.899)	0.426		
**M**					
M1/M0	3/241	4.438 (1.391–14.161)	0.012		
Mx/M0	73/241	1.440 (0.917–2.262)	0.113		
**Risk score**	317	6.630 (3.030–14.504)	<0.001	5.030 (2.248–11.254)	<0.001

TRIM, tripartite motif; TCGA-LIHC, The Cancer Genome Atlas Liver Hepatocellular Carcinoma.

### Correlations Between the Signature and Clinical Factors

**Figure 11:**
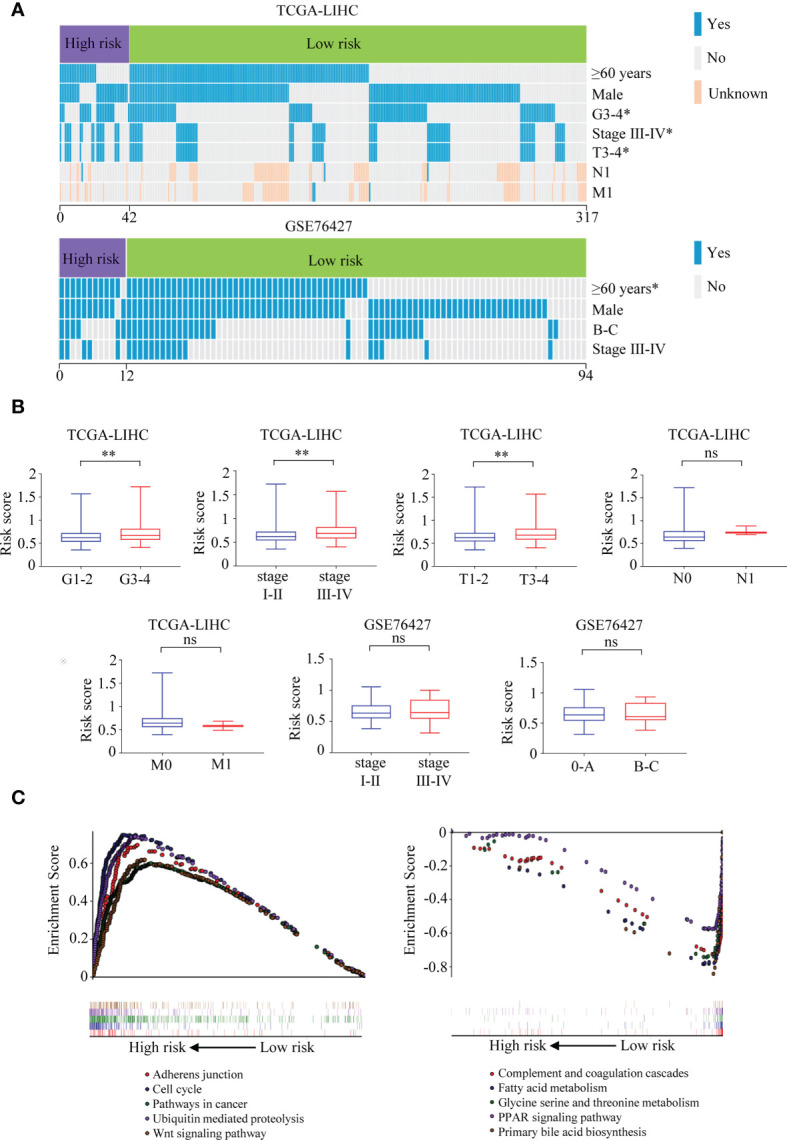
Correlations between the six-TRIM family gene risk score and clinical characteristics and GSEA for the signature. **(A)** Heatmaps describe the associations between the six-TRIM family gene risk score and clinical factors in TCGA-LIHC and GSE76427 cohorts. **(B)** Distributions and comparisons of the six-TRIM family gene risk score among different subgroups stratified by grade, TNM stage, T stage, N stage, and M stage in TCGA-LIHC cohort and TNM stage and BCLC stage in GSE76427 cohort. **(C)** GSEA shows the enriched KEGG pathways in the high- and low-risk groups based on the TRIM family gene-based signature. ns, no significance; *p < 0.05; **p < 0.01. TRIM, tripartite motif; GSEA, Gene Set Enrichment Analysis; TCGA-LIHC, The Cancer Genome Atlas Liver Hepatocellular Carcinoma; KEGG, Kyoto Encyclopedia of Genes and Genomes.

It was illustrated that the risk score was correlated with the clinical parameters in TCGA-LIHC. There was a higher percentage of the high-grade G3–4 cases in the high-risk group than in the low-risk group (52.4% vs. 35.6%, *p* = 0.037, **Figure 11A**, **Supplementary Table 2**). Similar results could be observed when referring to TNM stage (40.5% vs. 24.0%, *p* = 0.024) and T stage (38.1% vs. 23.6%, *p* = 0.045). In the GSE76427 cohort, there were more ≥60-year cases in the high-risk group than in the low-risk group (91.7% vs. 52.4%, *p* = 0.010, **Figure 11A**, **Supplementary Table 3**). What is more, the risk score was found to be roughly increased in patients with higher grade (*p* = 0.001), TNM stage (*p* = 0.002), and T stage (*p* = 0.003) in TCGA-LIHC (**Figure 11B**).

### Gene Set Enrichment Analysis

Comparing the high- and low-risk groups in TCGA-LIHC cohort (n = 343), Gene Set Enrichment Analysis (GSEA) was performed to explore the underlying mechanisms of the six-gene-based prognostic signature. The enriched KEGG pathways in the high-risk group were adherens junction, cell cycle, pathways in cancer, ubiquitin-mediated proteolysis, Wnt signaling pathway, and so on (**Figure 11C**). In the low-risk group, complement and coagulation cascades, patty acid metabolism, glycine serine and threonine metabolism, PPAR signaling pathway, primary bile acid biosynthesis, and other KEGG pathways were significantly enriched (**Figure 11C**).

### Establishment and Estimation of the TRIM Family Gene-Based Nomogram

**Figure 12:**
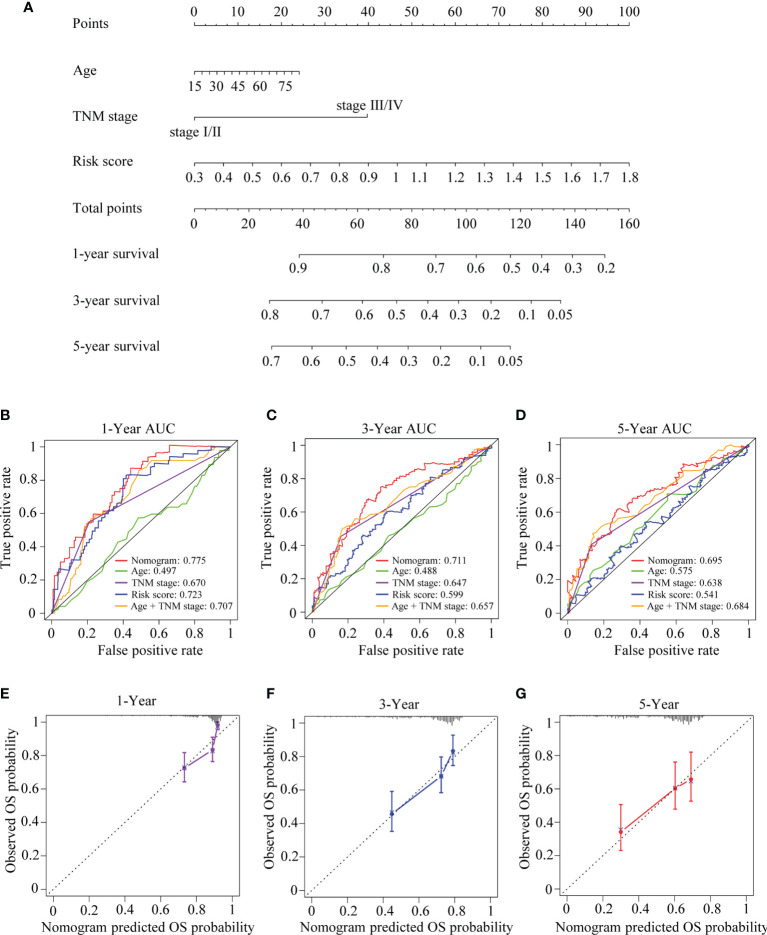
The developed nomogram for predicting OS in HCC. **(A)** The nomogram was built in TCGA-LIHC cohort with the six-TRIM family gene risk score, age, and TNM stage incorporated. **(B–D)** Time-dependent ROC curves for predicting 1- **(B)**, 3- **(C)**, and 5-year **(D)** OS based on the nomogram, age, TNM stage, risk score, or age + TNM stage in TCGA-LIHC cohort. **(E–G)** Calibration curves of the nomogram in terms of the agreement between predicted and observed 1- **(E)**, 3- **(F)**, and 5-year **(G)** OS. The relative 45° line represents an ideal prediction. Nomogram performance is indicated by the plot relative to the relative 45° line. TRIM, tripartite motif; OS, overall survival; HCC, hepatocellular carcinoma; TCGA-LIHC, The Cancer Genome Atlas Liver Hepatocellular Carcinoma; ROC, receiver operating characteristic.

Considering the results of the univariate and multivariate Cox regression analyses and clinical evidence of some parameters such as age and TNM stage for OS prediction, a nomogram was built by integrating risk score, age, and TNM stage to predict OS for patients with HCC (**Figure 12A**) based on TCGA-LIHC cohort. C-index, ROC curve, and calibration curve were applied to assess the discrimination and calibration for the nomogram. The C-index of the nomogram was 0.694, and the AUCs were 0.775 at 1 year, 0.711 at 3 years, and 0.695 at 5 years; and the largest AUCs for OS prediction were presented when compared with the age, TNM stage, risk score, and the age + TNM stage model (**Figures 12B–D**), suggesting that including the TRIM family signature to the model could provide improvement for OS prediction in HCC and present better discrimination. The calibration plots indicated that the nomogram performed well in comparison with an ideal model (**Figures 12E–G**).

## Discussion

HCC remains a global health issue, attracting wide attention due to its high incidence and mortality and poor outcomes. It is of great clinical significance to investigate the potential therapeutic targets and prognostic biomarkers, improve the outcome prediction, and facilitate the individualized clinical management for HCC.

TRIM family members have attracted more and more attention because of their important roles in cancer. Increasing evidence indicates that TRIM family proteins act as regulators of oncoproteins or tumor suppressor proteins, thus regulating cell growth, migration, metabolism, autophagy, and other biological processes (12, 54, 55). Their abnormal expressions and potential mechanisms have been confirmed in diverse cancers. Exploration of the biological and prognostic values of TRIM family proteins in cancers has flourished. Similar to our study, Xia et al. investigated the relation between the TRIM family and the prognosis of melanoma by bioinformatics analyses and discovered that TRIM27 might be a prognostic biomarker in melanoma (56). What is more, by using TRIM family genes, Wu et al. built a prognostic risk model by using TRIM family genes with a good predictive performance for kidney renal clear cell carcinoma, indicating the outcome predictive values of TRIM family in kidney renal clear cell carcinoma (57). Although some studies have indicated the significant prognostic roles of TRIM family members in HCC (22, 58, 59), the whole picture of the prognostic values of TRIM family members remains poorly characterized. Here, we tried to investigate the prognostic values of the TRIM family in HCC.

Our study was an attempt to explore the implications of the TRIM family members in HCC by analyzing the publicly available data. The hub TRIM family members were selected based on the results of the survival analysis and LASSO regression. A total of nine TRIM family members were identified as prognosis-associated genes in HCC, and six members were included in the TRIM family signature for risk stratification of HCC patients. Subsequently, a nomogram integrating the prognostic signature and clinical parameters was formulated to help us predict OS for HCC patients more intuitively. These results indicated that the TRIM family signature has a certain value in facilitating individualized treatment and clinical decision-making. Investigating the prognostic values of TRIM family members in HCC is important for the stratification of patients with diverse outcomes.

The human TRIM3 gene maps to chromosome 11p15.5, a region containing numerous cancer-related genes (60). It was validated that overexpression of TRIM3 could induce G0/G1 phase arrest, reducing cell proliferation in liver cancer (60). Chao et al. reported that decreased TRIM3 expression was associated with poor prognosis in HCC (59). However, the previous reports were not consistent with our findings as shown in **Figures 1B, C**, which suggested that HCC patients with high TRIM3 expression yielded poor survival. Since this article only collected the TRIM3 expression and HCC patient information from online databases, the role of TRIM3 in liver cancer needs to be verified with more databases, cell lines, clinical samples, and experimental researches. TRIM5, acting as a retrovirus limiting factor, serves a critical function in autophagic degradation, protecting immune cells from infection with HIV-1 (61). By using bioinformatics analysis, Wang et al. developed a prognostic model of glioma based on immune-related genes including TRIM5, suggesting its role in glioma (62). But the role of TRIM5 in HCC has not been uncovered clearly. MID1, a microtubule-associated ubiquitin E3 ligase, has been reported to play an important role in cancer. Zhang et al. revealed that MID1 functioned as an oncogene in lung adenocarcinoma, involved in apoptosis, proliferation, and cell cycle arrest (63). Besides, it was reported that MID1 could act as a translational inducer of AR protein, which in turn decreased MID1 levels in response to androgen stimulation in prostate cancer (64). TRIM21, well known to play a role in innate immunity, systemic lupus erythematosus, and Sjögren’s syndrome, has been observed to participate in cancer proliferation (65). It was demonstrated that TRIM21 was highly expressed in HCC, and genetic ablation of TRIM21 resulted in protection from oxidative hepatic damage and decreased carcinogenesis in HCC (66). However, Ding et al. found that downregulation of TRIM21 was correlated with poor prognosis in HCC (67). Given the different roles of TRIM21 in HCC suggested above, its mechanisms in HCC need to be explored with more experimental researches in the lab. TRIM27 was originally characterized as a gene related to oncogenic rearrangements with the RET proto-oncogene (68). It has been shown that TRIM27 had an oncogenic role in HCC cells by promoting cell proliferation, migration, and invasion (69). TRIM32 has been reported to be involved in cancer development. TRIM32 could regulate UVB-induced keratinocyte apoptosis through induction of nuclear factor-κB by promoting Piasy ubiquitination and degradation (70). TRIM32 could promote tumorigenesis *via* ubiquitination and degradation of Abi2 (71). In HCC, overexpression of TRIM32 could promote cell cycle progression, induce oxaliplatin resistance, and predict poor prognosis (72). TRIM44 may function as a “USP-like TRIM” and promote cancer development by regulating de-ubiquitination and stabilization of oncogenes, and its overexpression could contribute to malignant outcomes in gastric cancer (73). In HCC, it was validated that TRIM44 overexpression correlated with shorter OS; facilitated cell proliferation, migration, invasion; and enhanced resistance to doxorubicin (74). TRIM47 has been found as a mediator for malignant progression in multiple cancers (75, 76). In renal cell carcinoma, TRIM47 exerted an E3 ligase activity, binding to P53 protein to increase its ubiquitination and degradation of P53. In pancreatic cancer, TRIM47 could promote the aerobic glycolysis by interacting with and ubiquitination of fructose-1,6-bisphosphatase (FBP1). Some reports demonstrated that TRIM72 could act as a tumor suppressor in cancer. A recent report by Fernández-Aceñero et al. has demonstrated that low immunohistochemical expression of TRIM72 could predict relapse in stage II colon carcinoma (77). TRIM72 was also revealed to inhibit tumor progression in tongue cancer by regulating PI3K-AKT signaling pathway (78). However, the role of TRIM47 and TRIM72 in HCC is not yet clear, and investigating the mechanisms of TRIM47 and TRIM72 in HCC may shed light upon further biological values. Collectively, the TRIM family was worthy of further investigation in HCC, and the prognosis-related genes should be characterized for both their roles in tumor progression and their values as therapeutic targets.

However, there were some limitations in our study that need to be taken into consideration. First, our studies were based on public databases, and the sample size of the public datasets or collected human tissue samples was relatively limited, so larger population and multi-centered clinical studies are needed to increase the reliability of our results. Second, since all the mechanical analyses were descriptive in our study, more experiment researches in the lab were crucial for understanding the potential roles of the TRIM family in HCC. Third, more clinical parameters concerning the progression and prognosis of HCC such as the presence of cirrhosis, Child-Pugh scoring, vascular invasion, or other parameters should be included to better understand the association between the TRIM family and HCC. Due to the limitations above, further well-designed studies are required to increase the credibility of our findings.

## Conclusion

We systemically explored the roles of the TRIM family in HCC by a series of bioinformatics analyses. Our results may contribute to finding novel prognostic biomarkers and developing new therapeutic targets.

## Data Availability Statement

Publicly available datasets were analyzed in this study. These data can be found here: TCGA database: https://portal.gdc.cancer.gov/; GEO database: https://www.ncbi.nlm.nih.gov/geo/.

## Ethics Statement

The studies involving human participants were reviewed and approved by the Medical Ethics Committee of Nanfang Hospital, Southern Medical University. The patients/participants provided their written informed consent to participate in this study.

## Author Contributions

JidW, SL, JJL, and AL contributed to the concept and design of the study. WD, YX, JYL, LH, and WT performed the data collection, data analyses, and experiments in the lab. MP, JZ, PY, and XW carried out the statistical analyses. JinW, ZW, and AL collected the human samples. XJ and PJ coordinated the project. LX and AL supervised the project. WD wrote and revised the manuscript. All authors contributed to the article and approved the submitted version.

## Funding

This work was supported by grants from the National Natural Science Foundation of China (Nos. 81772964, 81974448, and 82073066), the President Foundation of Nanfang Hospital, Southern Medical University (No. 2020C005), National Major New Drug Creation Science and Technology Major Special Fund Funding Project (No. 2020ZX09201017), Guangdong gastrointestinal disease research center (No. 2017B020209003), the Special Scientific Research Fund of Public Welfare Profession of National Health and Family Planning Commission (No. 201502026), and Shenzhen Science and Technology Innovation Commission (Nos. JCYJ20180306170328854 and JCYJ20210324135005013).

## Conflict of Interest

The authors declare that the research was conducted in the absence of any commercial or financial relationships that could be construed as a potential conflict of interest.

## Publisher’s Note

All claims expressed in this article are solely those of the authors and do not necessarily represent those of their affiliated organizations, or those of the publisher, the editors and the reviewers. Any product that may be evaluated in this article, or claim that may be made by its manufacturer, is not guaranteed or endorsed by the publisher.

## Supplementary Material

Supplementary Figure 1 Univariate Cox regression analyses of the five prognostic biomarkers (PDCD10, TFAP4, LYRM4, VPS35 and PPM1D) in TCGA-LIHC **(A)** and GSE76427 cohorts **(B)**.

Supplementary Figure 2 Risk score analyses including the risk score distributions, survival statuses and gene expression levels. **(A, B)** The profiles of the risk score distributions, survival statuses and heatmaps of gene expression levels in TCGA-LIHC **(A)** and GSE76427 **(B)**.

Supplementary Table 1 List of the primers used for qRT-PCR.

Supplementary Table 2 Clinical characteristics of the TCGA-LIHC cohort patients in different risk groups based on the TRIM family gene-based signature.

Supplementary Table 3 Clinical characteristics of the GSE76427 cohort patients in different risk groups based on the TRIM family gene-based signature.
