# Supplementary figures and images for: Comprehensive Analysis of the Prognostic Values of the TRIM Family in Hepatocellular Carcinoma
Source: Front Oncol. 2021 Dec 23;11:767644. doi: 10.3389/fonc.2021.767644 (PMC8733586; doi:10.3389/fonc.2021.767644)

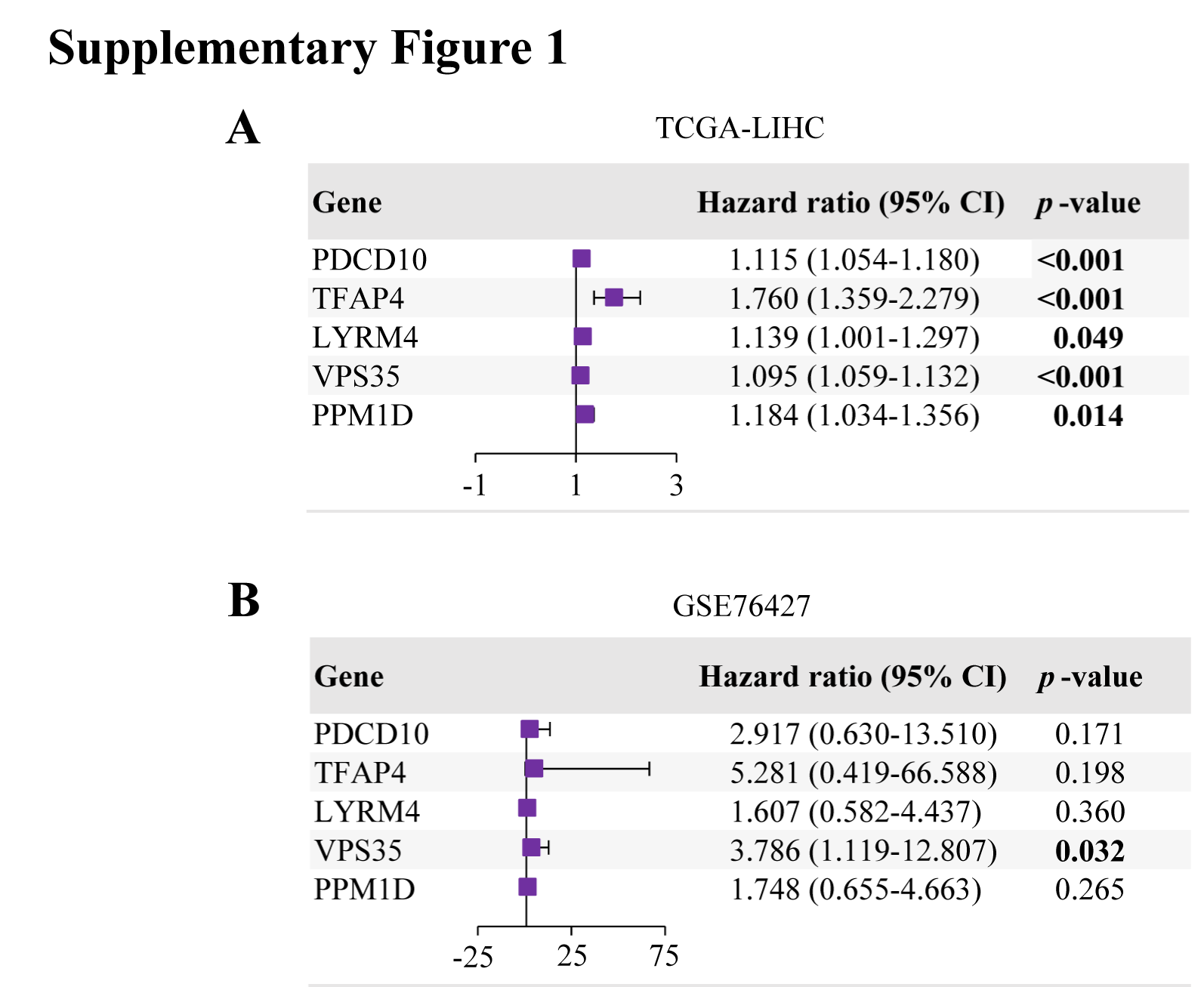

Supplement: Supplementary Figure 1 — Univariate Cox regression analyses of the five prognostic biomarkers (PDCD10, TFAP4, LYRM4, VPS35 and PPM1D) in TCGA-LIHC (A) and GSE76427 cohorts (B). [file Image_1.tif]

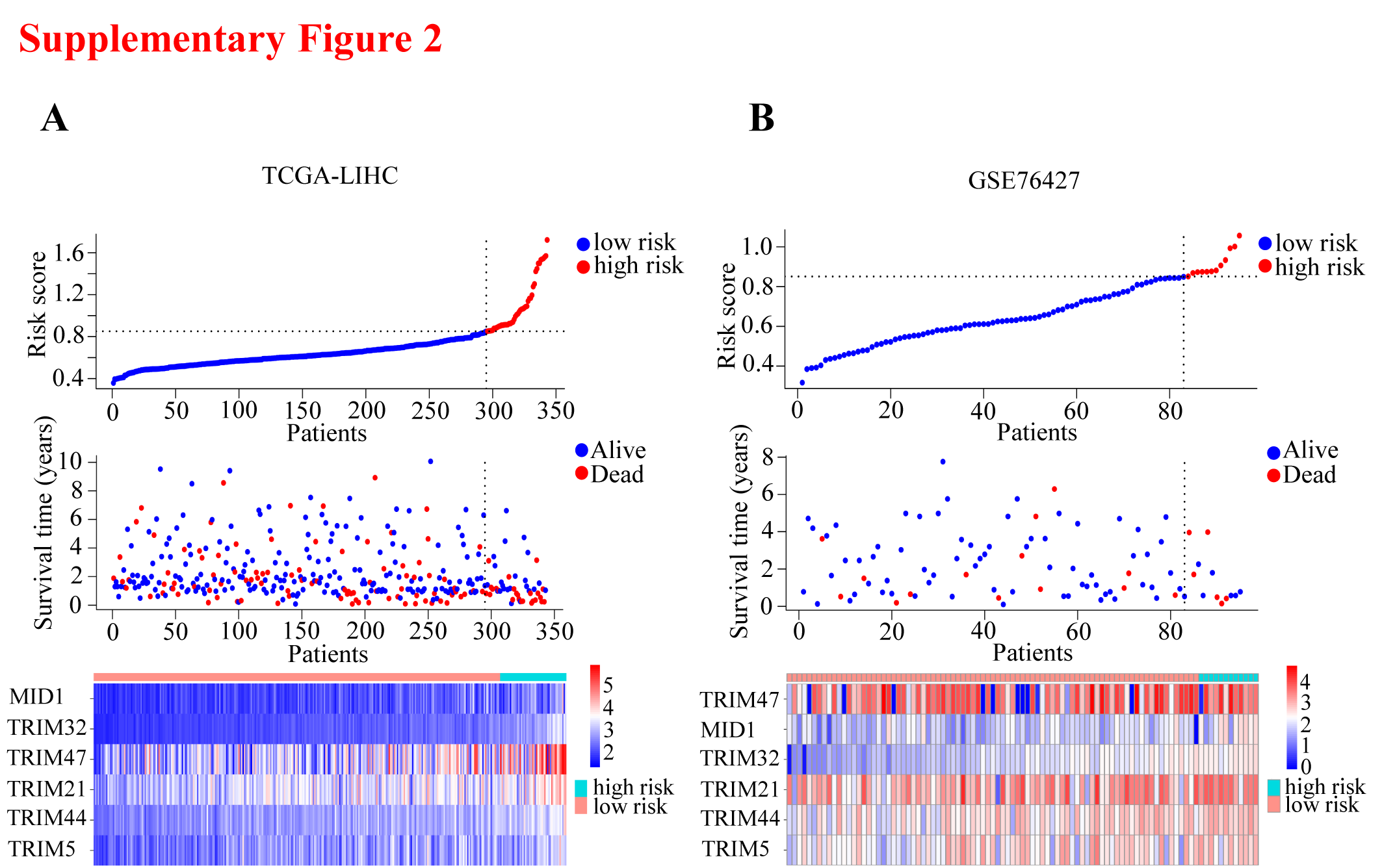

Supplement: Supplementary Figure 2 — Risk score analyses including the risk score distributions, survival statuses and gene expression levels. (A, B) The profiles of the risk score distributions, survival statuses and heatmaps of gene expression levels in TCGA-LIHC (A) and GSE76427 (B). [file Image_2.tif]
