# Supplementary material for: Comprehensive Analysis of the Prognostic Values of the TRIM Family in Hepatocellular Carcinoma
Source: Front Oncol. 2021 Dec 23;11:767644. doi: 10.3389/fonc.2021.767644 (PMC8733586; doi:10.3389/fonc.2021.767644)
Supplement: Supplementary Table 1 — List of the primers used for qRT-PCR. [file Table_1.docx]

**Supplementary Table 1.** List of the primers used for qRT-PCR.

| **Name** | **Position or orientation** | **Sequence (5’-3’)** |
| --- | --- | --- |
| TRIM3 | F | GCGACCTGGAGACCATTTGT |
|  | R | GCTACTGCCGATGTGTTCCTG |
| TRIM5 | F | AAGTCCATGCTAGACAAAGGAGA |
|  | R | GTTGGCTACATGCCGATTAGG |
| MID1 | F | CTGACCTGCCCTATTTGTCTG |
|  | R | GCACAGTGTGATACTAGGATGC |
| TRIM21 | F | TCAGCAGCACGCTTGACAAT |
|  | R | GGCCACACTCGATGCTCAC |
| TRIM27 | F | AGCCCATGATGCTCGACTG |
|  | R | GGGCACGACACGTTAGTCT |
| TRIM32 | F | CCGGGAAGTGCTAGAATGCC |
|  | R | CAGCGGACACCATTGATGCT |
| TRIM44 | F | CCATCTGGCCGAATACGTCC |
|  | R | TGCCTCGCTTTCTATCTCCCT |
| TRIM47 | F | CTGAGCAGTCCAAAGTCCTGA |
|  | R | CTACGGCTGCACTCTTGATG |
| TRIM72 | F | CGTCCTCTCCTTCTACGATG |
|  | R | CATTCTTGCCCTTGTCGTG |
| GAPDH | F | AAATCCCATCACCATCTTCC |
|  | R | TCACACCCATGACGAACA |
