# Supplementary material for: Comprehensive Analysis of the Prognostic Values of the TRIM Family in Hepatocellular Carcinoma
Source: Front Oncol. 2021 Dec 23;11:767644. doi: 10.3389/fonc.2021.767644 (PMC8733586; doi:10.3389/fonc.2021.767644)
Supplement: Supplementary Table 2 — Clinical characteristics of the TCGA-LIHC cohort patients in different risk groups based on the TRIM family gene-based signature. [file Table_2.docx]

**Supplementary Table 2.** Clinical characteristics of the TCGA-LIHC cohort patients in different risk groups based on the TRIM family gene-based signature.

| **Characteristics** | **Whole cohort (n=317)** | **Low risk (n=275)** | **High risk (n=42)** | ***p*-value** |
| --- | --- | --- | --- | --- |
| **Age** |  |  |  |  |
| ＜60 years | 151(47.6%) | 131(47.6%) | 20(47.6%) | 0.998 |
| ≥60 years | 166(52.4%) | 144(52.4%) | 22(52.4%) |  |
| **Gender** |  |  |  |  |
| Female | 99(31.2%) | 88(32.0%) | 11(26.2%) | 0.449 |
| Male | 218(68.8%) | 187(68.0%) | 31(73.8%) |  |
| **Grade** |  |  |  |  |
| G1-2 | 197(62.1%) | 177(64.4%) | 20(47.6%) | **0.037** |
| G3-4 | 120(37.9%) | 98(35.6%) | 22(52.4%) |  |
| **TNM stage** |  |  |  |  |
| I-II | 234(73.8%) | 209(76.0%) | 25(59.5%) | **0.024** |
| III-IV | 83(26.2%) | 66(24.0%) | 17(40.5%) |  |
| **T** |  |  |  |  |
| T1-2 | 236(74.4%) | 210(76.4%) | 26(61.9%) | **0.045** |
| T3-4 | 81(25.6%) | 65(23.6%) | 16(38.1%) |  |
| **N** |  |  |  |  |
| N0 | 238(75.1%) | 203(73.8%) | 35(83.3%) | 0.133 |
| N1 | 3(0.9%) | 2(0.7%) | 1(2.4%) |  |
| Nx | 76(24.0%) | 70(25.5%) | 6(14.3%) |  |
| **M** |  |  |  |  |
| M0 | 241(76.0%) | 208(75.6%) | 33(78.6%) | 0.900 |
| M1 | 3(0.9%) | 3(1.1%) | 0(0.0%) |  |
| Mx | 73(23.0%) | 64(23.3%) | 9(21.4%) |  |
