# Supplementary material for: Comprehensive Analysis of the Prognostic Values of the TRIM Family in Hepatocellular Carcinoma
Source: Front Oncol. 2021 Dec 23;11:767644. doi: 10.3389/fonc.2021.767644 (PMC8733586; doi:10.3389/fonc.2021.767644)
Supplement: Supplementary Table 3 — Clinical characteristics of the GSE76427 cohort patients in different risk groups based on the TRIM family gene-based signature. [file Table_3.docx]

**Supplementary Table 3.** Clinical characteristics of the GSE76427 cohort patients in different risk groups based on the TRIM family gene-based signature.

| **Characteristics** | **Whole cohort (n=94)** | **Low risk (n=82)** | **High risk (n=12)** | ***p*-value** |
| --- | --- | --- | --- | --- |
| **Age** |  |  |  |  |
| ＜60 years | 40(42.6%) | 39(47.6%) | 1(8.3%) | **0.010** |
| ≥60 years | 54(57.4%) | 43(52.4%) | 11(91.7%) |  |
| **Gender** |  |  |  |  |
| Female | 12(12.8%) | 11(13.4%) | 1(8.3%) | 1.000 |
| Male | 82(87.2%) | 71(86.6%) | 11(91.7%) |  |
| **TNM stage** |  |  |  |  |
| I-II | 72(76.6%) | 65(79.3%) | 7(58.3%) | 0.144 |
| III-IV | 22(23.4%) | 17(20.7%) | 5(41.7%) |  |
| **Bclc stage** |  |  |  |  |
| 0-A | 59(62.8%) | 53(64.6%) | 6(50.0%) | 0.353 |
| B-C | 35(37.2%) | 29(35.4%) | 6(50.0%) |  |
